# Anthelmintic Treatment Does Not Change Foraging Strategies of Female Eastern Grey Kangaroos, *Macropus giganteus*

**DOI:** 10.1371/journal.pone.0147384

**Published:** 2016-01-19

**Authors:** Jemma K. Cripps, Jennifer K. Martin, Graeme Coulson

**Affiliations:** 1 School of BioSciences, The University of Melbourne, Melbourne, Victoria, Australia; 2 Faculty of Veterinary Science, The University of Melbourne, Veterinary Clinical Centre, Werribee, Victoria, Australia; Universidade de Aveiro, PORTUGAL

## Abstract

Large mammalian herbivores are commonly infected with gastrointestinal helminths. Heavily parasitised hosts are likely to have increased nutritional requirements and would be predicted to increase their food intake to compensate for costs of being parasitised, but experimental tests of the impacts of these parasites on the foraging efficiency of hosts are lacking, particularly in free-ranging wildlife. We conducted a field experiment on a population of free-ranging eastern grey kangaroos (*Macropus giganteus*) to test this prediction, removing nematodes from one group of adult females using an anthelmintic treatment. We then carried out observations before and following treatment to assess the influence of parasites on foraging behaviour. Contrary to our predictions, the manipulation of parasite burdens did not result in changes in any of the key foraging variables we measured. Our results suggest that despite carrying large burdens of gastrointestinal parasites, the foraging strategy of female kangaroos is likely be driven by factors unrelated to parasitism, and that kangaroos in high nutritional environments may be able acquire sufficient nutrients to offset the costs of parasitism. We conclude that the drivers of forage intake likely differ between domesticated and free-ranging herbivores, and that free-ranging hosts are likely more resilient to parasitism.

## Introduction

Mammalian herbivores must make complex decisions whilst foraging [1]. These decisions may be influenced by biotic factors, such as the quality and quantity of resources [2], as well as the distribution of these resources within the environment [3]. In addition, herbivores must balance foraging efficiency with numerous constraints, including predation risk [4, 5], conspecific competition for resources [6] and ingestion of plant toxins [7]. One constraint that has received inadequate attention in herbivorous foraging contexts is the impact of parasites, in particular, gastrointestinal nematodes that commonly infect mammalian herbivores. Most of these gastrointestinal nematodes are directly transmitted via the faecal-oral route [8]. Hosts release eggs in their faeces, which hatch into infective larvae, before migrating onto herbage and being ingested by other hosts as they graze in the surrounding area. Herbivorous hosts might therefore alter their foraging strategy in response to their parasite burdens, as a behavioural defence to avoid ingesting more parasites or to compensate for parasitic costs. However these possibilities are rarely examined in a free-ranging context using behavioural sampling and robust experimental design.

Although most mammals tend to develop some immunity to gastrointestinal parasites [9], parasites often cause significant disease, particularly in individual hosts experiencing nutritional stress [10]. This is primarily due to the pathological changes that occur within the gastrointestinal tract, disrupting digestion and nutrient intake [11, 12]. However, more subtle subclinical effects have also been described [13]. Most research into these effects has focused on domestic or semi-domestic ruminants, where pervasive subclinical impacts are possibly more conspicuous [13] and the effects are relatively well understood; the resulting reductions in body condition [14], growth [15], and reproduction [16] engender production losses worldwide [17]. However, subclinical effects are recognised in some wildlife hosts [18, 19], with reported impacts including reductions in body condition [20] and fecundity [21, 22].

Modifications to host foraging behaviour are predicted to evolve when parasites have detrimental impacts [23]. Parasite avoidance has probably received the most attention in foraging contexts [24, 25]. Avoidance can occur on a broad scale, with shifts in foraging within a home range [26], or at a fine scale, where foraging patches contaminated with faeces are avoided [27–29]. Foraging in close proximity to faeces carries an increased risk of infection with both microparasites, e.g. bacterial pathogens [30] and macroparasite larvae, which have limited mobility and tend to be distributed close to faecal deposits [31]. However, grazing decisions could also be affected by a host’s physiological state. For example, a heavily parasitised host is likely to have increased nutritional requirements and might increase its food intake to compensate for the costs of being parasitised. Evidence in domestic and semi-domestic herbivores suggests that individuals monitor their physiological state (e.g. elevated metabolic rate and hypoalbuminaemia [11]) and can change their foraging behaviour accordingly [28, 32–34]. Nematode parasitism likely imposes energetic costs on hosts, due to reduced absorption of nutrients and the maintenance of a competent immune system [35, 36]. Any reductions in parasite levels would therefore be predicted to reduce pressure on a host to increase energy intake, as a means of compensating for these costs [36]. The few studies of wildlife hosts that have investigated how foraging is affected by individual differences in physiological state have used indirect measures of parasite burdens (such as faecal egg counts), rather than rigorous experimental manipulations [37, 38]. Experimental approaches using antiparasitic treatments are now more widely used as a method to quantify the impacts of infection [39]

We investigated the effect of parasites on the foraging behaviour of free-ranging eastern grey kangaroos (*Macropus giganteus*) by experimentally manipulating parasite burdens under natural conditions. Eastern grey kangaroos carry a diverse fauna of gastrointestinal nematode parasites [40], with most species showing seasonal fluctuations, with burdens peaking in the winter months [41]. Populations of eastern grey kangaroos can reach high densities, and individuals are gregarious, forming mixed-sex, open-membership groups to forage and rest [42], conditions that favour helminth parasite transmission [43]. Unlike many other herbivores, eastern grey kangaroos do not exhibit localised defaecation, rather they defaecate randomly throughout foraging areas [44]. A study by Garnick *et al*. [27] demonstrated that eastern grey kangaroos exhibit faecal aversion behaviour, and do not accept a higher risk of parasitism for increased nutrient trade-off. There was limited scope for investigating faecal aversion in our system, as the population was at high density and the pasture heavily contaminated with faeces. Consequently, we chose to focus primarily on other measures of foraging behaviour. We also chose to focus solely on adult female kangaroos to reduce potential influences of sex and body size on parasite burdens [45], and because the risk of infection may be greatest for reproducing females, due to increases in food intake during lactation [46]. We hypothesised that kangaroos adjust foraging rates as a mechanism to compensate for the costs of parasitism, as heavily parasitised hosts are likely to have increased nutritional requirements. No data exists for marsupial hosts so we predicted that experimental reductions in parasite burdens of female kangaroos would result in decreased feeding motivation, expressed as decreases in four key foraging variables: proportion of time foraging, proportion of time chewing, bite rate, and movement through foraging patches.

## Materials and Methods

### Study site

This experiment was conducted at the Anglesea Golf Club (38°24’S, 114°10’E) in southern Victoria, Australia in July–September 2011. All data was collected with land owner (The Anglesea Golf Club) consent, and all manipulations occurred in strict accordance with permission granted by The University of Melbourne’s Animal Ethics Committee (project 1011709) and the Department of Sustainability and Environment (research permit 10005557). Kangaroos were captured using a well-established capture technique for habituated kangaroos, and were sedated with Zoletil® 100 at a dose of approximately 5 mg/kg body mass (full details provided in Methods) in accordance with Australian Government National Health and Medical Research Council (NHMRC) guidelines. This field study did not involve endangered or protected species. The golf course covers an area of 73 ha and contains open, grassy fairways dominated by couch grass (*Cynodon dactylon*), separated by patches of woodland and shrubland [47]. The course is bordered by native heathy woodland to the north and west; kangaroos move freely between the course and native vegetation, as well as through residential properties in the south and east. Population surveys at the time of the study showed that the population density of kangaroos at the site was approximately 2/ha [47]. High fecundity was observed during the study period [47]. Potential predators at the site include the red fox (*Vulpes vulpes*) and domestic dogs (*Canis lupus familiaris*). Counts of infective-stage larvae showed that mean (± SE) levels within the environment during July-August 2011 were 2121 ± 804 larvae per kg dry weight of pasture [41]. Gastrointestinal parasites were examined from ten individuals found dead at the site, May 2010 –August 2011 [41]. Necropsies revealed a diverse gastrointestinal parasite community, including 17 species of nematode (*Rugopharynx macropodis*, *R*. *rosemariae*, *Pharyngostrongylus kappa*, *Cloacina pelops*, *C*. *herceus*, *C*. *hermes*, *C*. *selene*, *C*. *artemis*, *C*. *expansa*, *C obtuse*, *Alocostoma clelandi*, *Labiosimplex kungi*, *L*. *bipapillosus*, *Globocephaloides trifidospicularis*, *Macropoxyuris brevigularis*, *M*. *longigularis* and *Macropostrongyloides baylisi)* and one cestode species (*Progamotaenia festiva)*.

### Experimental design

Due to their habituation to humans, kangaroos at Anglesea tolerate a close approach and can be captured, marked and observed with relative ease. Consequently, the urban kangaroo population at Anglesea has been the focus of a number of studies of their ecology [48, 49] and management [47, 50, 51], and during the period of our study, over 130 tagged individuals were present in the population. From July to September 2011, 242 observations were collected from 25 adult female kangaroos, each individually identifiable with a collar and unique combination of coloured, reflective ear tags. Only reproducing, adult female kangaroos were observed to avoid any impacts of sex and reproductive status on foraging strategies [46, 52] and/or parasite resistance [53]. Behavioural observations were divided into two periods: (1) before experimental parasite manipulation (7 weeks, July–August 2011, 122 observations) and (2) after parasite manipulation (7 weeks, August–September 2011, 120 observations). This allowed a Before-After-Control-Impact (BACI) design to be used [54].

### Capture and Treatment

Kangaroos were captured in August 2011 using a telescopic pole syringe (1.4, 2.4 or 3.6 m long) [55]. Animals were approached on foot and injected in the hind limb musculature with Zoletil® 100 (100 mg/mL of 50:50 tiletamine hydrochloride-zolazepam hydrochloride mixture; Virbac Animal Health Pty Ltd, Milperra, New South Wales, Australia) at a dose of approximately 5 mg/kg body mass. Standard body measurements [56] were collected using a retractable tape measure and Vernier calipers. Leg, pes and arm lengths were measured to the nearest mm; body mass was measured to the nearest 0.5 kg using 50-kg spring scales (Salter, Melbourne, Victoria, Australia). For females with pouch young (*n* = 24), sex of the young was recorded.

Parasite burdens in female kangaroos were experimentally manipulated using an anthelmintic drug treatment. Individuals were randomly allocated to either a control (*n* = 13) or a treatment (*n* = 12) group. Treated individuals were given an oral dose of albendazole (Alben® for sheep, lambs and goats, 19 g/L, Virbac Animal Health Pty Ltd, Milperra, New South Wales, Australia) at a rate of 3.8 mg/kg body mass [57], creating a Low parasite burden group. Kangaroos remained free-ranging for the duration of the experiment and continued to forage on contaminated pasture following treatment. The High parasite burden group comprised control individuals, which were left untreated, with naturally occurring helminth burdens. No oral control was administered to untreated individuals to avoid indirectly affecting the gastrointestinal fauna. Albendazole has 100% efficacy in eastern grey kangaroos [57]. At the time of treatment, there were no differences between groups in body mass (Low: 27.5 ± 0.65 kg, High: 26.78 ± 0.61 kg).

We based the timing of our post-treatment observation period (1–1.5 weeks following anthelmintic treatment) on the information available for domestic herbivores since no equivalent data were available on wildlife hosts. Studies of cattle *Bos primigenius* [58] and sheep *Ovis aries* [34] have shown that hosts adjusted their behaviour within a week of parasite treatment.

### Foraging behaviour

Focal animal sampling [59] was used to investigate the fine-scale foraging behaviour of kangaroos. During both observations periods, focal samples were carried out at dawn and dusk, when eastern grey kangaroos forage most actively [60, 61], and observations took place only when more than half of the visible kangaroos were foraging rather than resting. Focal females were selected using stratified random sampling. Focal kangaroos were recorded for 4–6 min on a high-definition video camera (Panasonic SD40, Panasonic Australia Pty. Ltd.), attached to an extendable tripod (1.6–2 m, Bushnell Australia). The kangaroos were not disturbed by observations on foot from a distance of 6–23 m. If the focal kangaroo moved out of sight or was obviously interrupted by the presence of golfers or dogs (ascertained by the level and direction of vigilance), the observation was discarded and the individual resampled at a later time. Observations that lasted for less than 4 min were not used in the analysis.

For each observation, the camera was positioned so that the focal female was foraging with her head facing towards the camera, with her mouth visible. Each time a focal kangaroo took a step that changed her direction of foraging, the camera was moved so that it was again positioned head-on. Behavioural events [59] recorded whilst the kangaroo foraged included the number of bites and steps taken. Behavioural states [59] that were recorded were the proportion of time spent feeding, chewing and vigilant. Chewing and foraging were calculated as two independent behaviours, as chewing allowed individuals to process food whilst maintaining vigilance. While kangaroos can simultaneously take steps or bites whilst foraging, activities such as vigilance and foraging are mutually exclusive behaviours.

For each female selected, the date, time, pasture height, reproductive class, group size, and the distance to her nearest neighbour (both at the beginning and end of the focal observation) were recorded. Pasture height was allocated to one of three categories: (1) heavily mown grass on the fairways and greens (5.9 ± 0.94 mm), (2) heavily grazed grass on the edge of the fairways (26.5 ± 2.65 mm), and (3) long grass patchily distributed in woodland and residential properties (88.8 ± 6.8 mm). Pasture measurements were based on 10 samples collected for each category. Reproductive state was determined based on the presence or absence of pouch young. A group of kangaroos was classified as all individuals within 30 m of their nearest neighbour [62]; group size equaled 1 for solitary individuals. Distances between individuals were estimated based on the body lengths of females [63].

Four key foraging variables were calculated: the total proportion of time feeding (a measure of an individual’s trade-off between its energetic requirements, vigilance and maternal care, after Maguire *et al*. [64]), the gross bite rate and proportion of time chewing (measures of intake and initial processing of forage, respectively, after Ruckstuhl *et al*., [65] and Maguire *et al*. [64]), and bite-step ratio (a measure of foraging intensity in patches, high values indicating intense, non-selective foraging, after Garnick *et al*. [27]). In total, 4–5 focal samples were collected for each of the 12 parasite-treated females and 13 control females before and following anthelmintic treatment (S1 Dataset).

### Faecal egg counts

Faecal samples were collected fresh from each individually-identifiable female in 2-h periods around dawn and dusk, when kangaroos were actively foraging and defecation rates were greatest [44]. Due to the habituation of the kangaroos on the golf course, individuals tolerated a close approach and could be observed closely until they defecated. The observer could collect faecal samples immediately after deposition, simply by walking to the point of defacation on the pasture. Samples were stored at 4°C and processed within two days of collection. A total of 75 faecal samples were collected from the 25 study kangaroos (S2 Dataset).

In the laboratory, faecal samples were analysed and the number of eggs per gram (epg) were determined by a modified McMaster technique. 2 g of faeces were mixed with 60 mL of saturated sodium nitrate solution (Redox Pty Ltd, Minto, New South Wales, Australia). An aliquot of 0.5 mL of homogenized filtrate was transferred into a Whitlock Universal counting chamber, before being examined under a microscope at 100x magnification. Strongylid eggs, which were thin-shelled and ellipsoidal were counted, with each egg representing 60 epg of faeces. It is not possible to distinguish the eggs of the various cloacinid nematodes of kangaroos, so the egg counts could not be discriminated for different taxa. No cestode eggs were detected in the faecal flotations. *Eimeria* (Phylum Apicomplexa) oocysts were present in some samples but at very low numbers.

### Statistical analysis

Strongyle nematode egg counts were log (1+epg) transformed to meet the assumptions of normality. Pre-treatment egg counts were analysed using ANOVA. Analysis of the effects of treatment on kangaroo faecal egg counts was carried out using restricted maximum-likelihood analyses (REML), with time and treatment as fixed factors, and kangaroo identity as a random factor to account for repeated measures. Statistical analyses were carried out using Genstat, Version 10 (VSN International Ltd., Hemel Hempstead, UK) and results were considered significant at *P* ≤ 0.05. Faecal egg count reduction calculations were made according to Wood *et al*. [66] using the Excel plug-in ‘Reso’ [67].

Analysis of foraging behaviour was conducted using the behavioural software JWatcher v.1.0 [68], which allowed each behaviour to be coded whilst accounting for the proportion of time the focal animal was out of sight. Linear mixed models were used to analyse foraging and chewing durations, bite rate and step-bite ratio, in the program SPSS Version 21 (IBM Corporation, Armonk, New York, USA). Individuals were included as random factors. Linear mixed models allowed the inclusion of extrinsic factors, such as time of day, observation period (before or after treatment), group size, nearest-neighbour distance, pasture category and temperature, and intrinsic factors, such as treatment group, and sex of pouch young, which is known to influence the foraging behaviour of kangaroos [48, 52, 69]. Results were considered significant at *P* ≤ 0.05. Correlations between all the variables were explored. Bite rate and chewing duration were not significantly correlated (*r* = 0.07, *P* = 0.25), nor was the nearest-neighbour distance at the start of the focal sample and group size (*r* = -0.11, *P* = 0.09). The nearest-neighbour distance at the beginning and end of the focal observation were highly negatively correlated (*r* = -0.79, *P* < 0.01) so the nearest-neighbour distance at the end of the observation was excluded from the analysis. The models were run with main effects and the interaction between treatment and observation period. Although this analysis tested multiple statistical hypotheses, the treatment by observation period interaction was of primary interest to investigate the treatment effect. Bite rate was included as a covariate in the model for chewing duration to account for the effects of food intake.

## Results

### Faecal Egg counts

**Fig 1:**
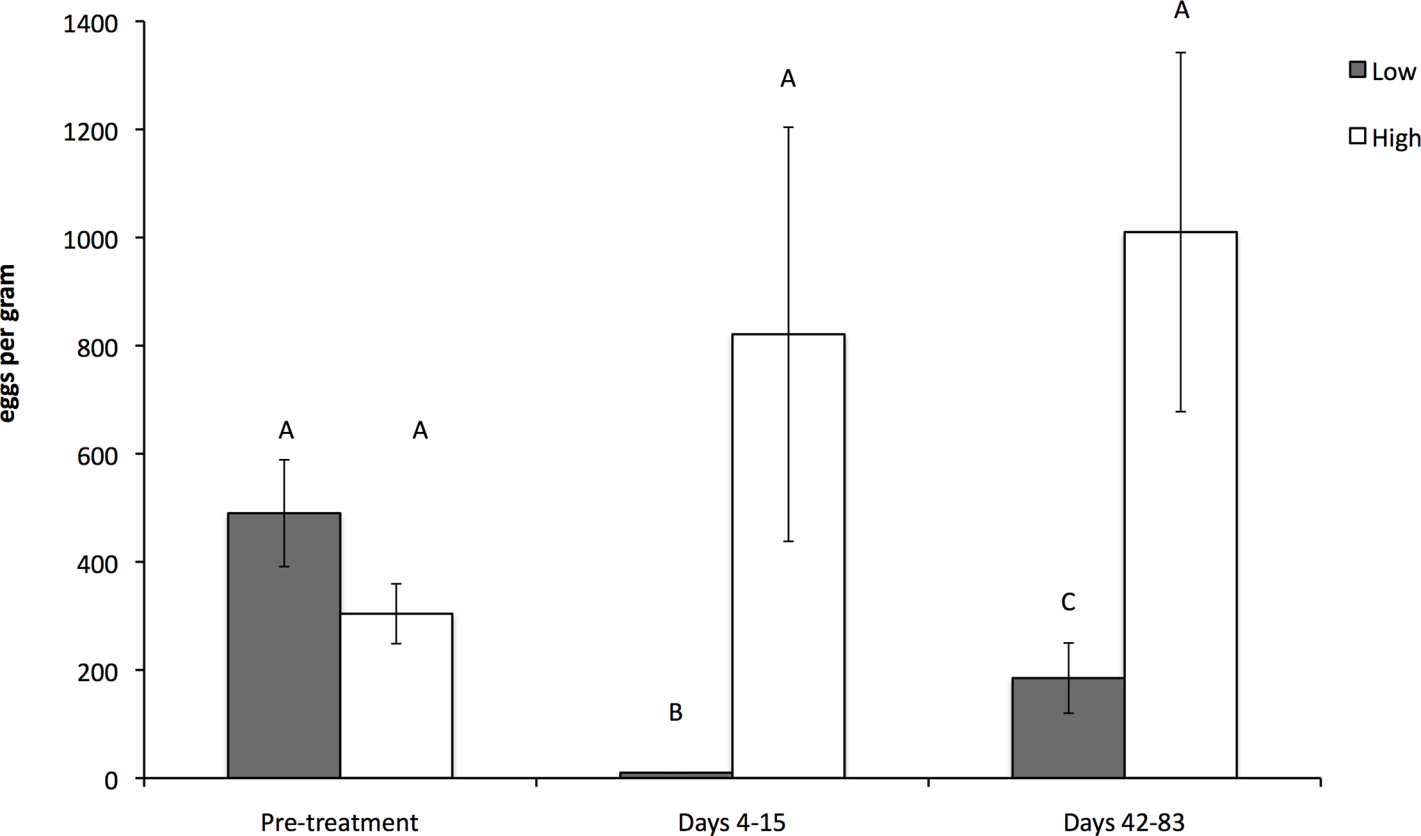
Faecal egg counts. Mean faecal egg counts for female eastern grey kangaroos with Low and High parasite burdens in three periods: pre-treatment, post-treatment 4–15 days and post-treatment 42–83 days, at the Anglesea Golf Club, Victoria, Australia from July to August 2011. Error bars indicate standard error. Columns not labelled with the same letter are significantly different.

Prior to anthelmintic treatment, there was no difference in strongyle faecal egg counts between the Low and High nematode burden groups (*F*_1,23_ = 1.56, *P* = 0.22, Fig 1). Following treatment, mean (± SE) faecal egg counts of females in the Low parasite group (0 ± 0 epg) were significantly lower than those females in the High parasite group (821 ± 383 epg, *F*_1,23_ = 94.54, *P* < 0.001, Fig 1), resulting in a 100% reduction in faecal egg counts. There was a significant interaction between time and treatment (*F*_1,23_ = 217.54, *P* < 0.001), such that faecal egg counts in the Low group increased more than those in the control group, but egg counts in the Low group were still significantly lower days 42–83 post-treatment. No cestode eggs were detected in the faecal flotations. *Eimeria* oocysts were present in some samples but at very low numbers.

### Proportion of time spent feeding

**Fig 2:**
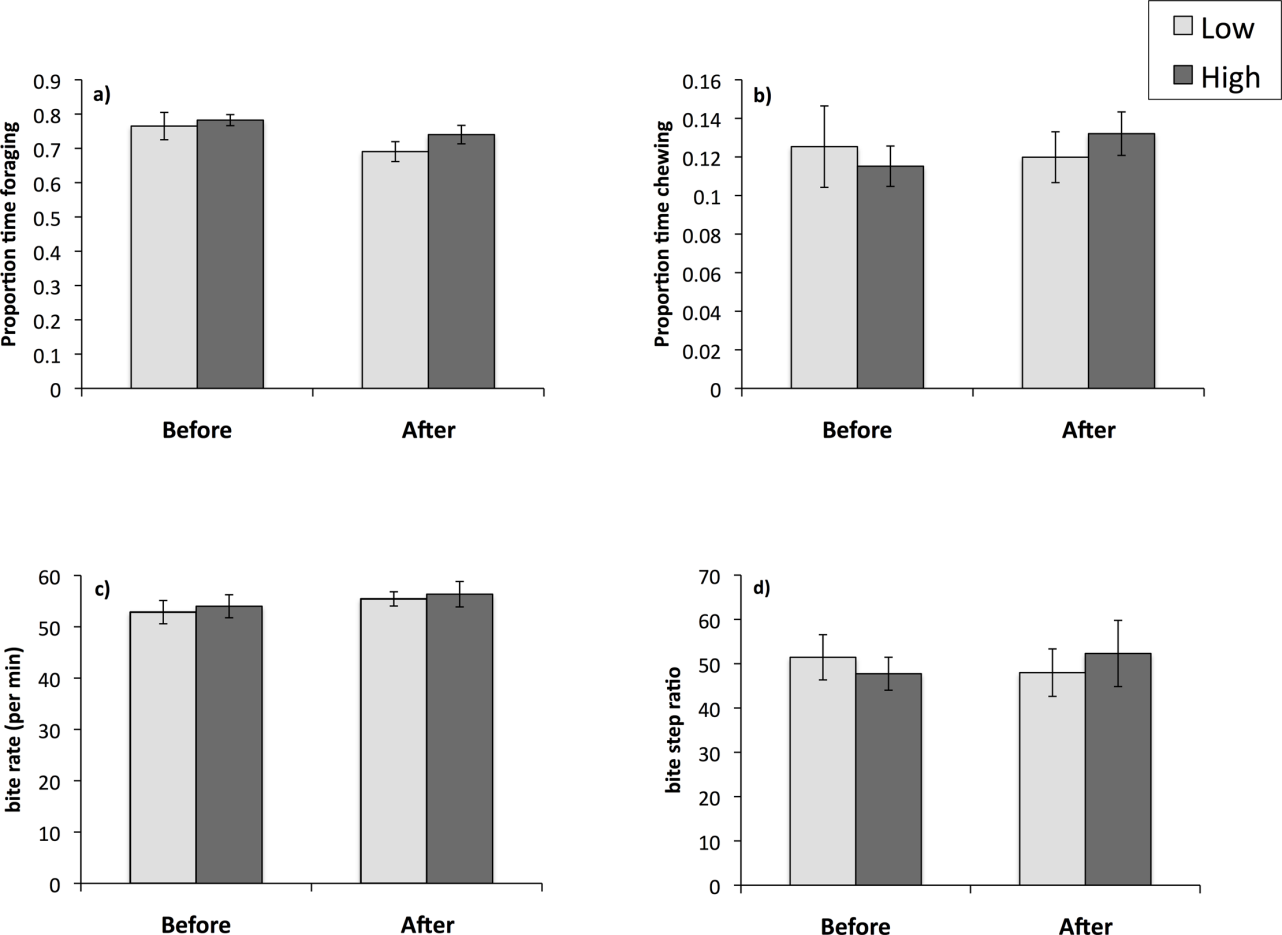
Anthelmintic treatment and foraging behavior. The effect of parasite burden on a) the proportion of time foraging, b) the proportion of time chewing, c) the bite rate and d) the bite step ratio of female eastern grey kangaroos before and after treatment at Anglesea Golf Club, Victoria, Australia. Kangaroos in the Low group were treated with albendazole (*n* = 12), while kangaroos in the High group remained untreated (*n* = 13). Error bars indicate standard error.

Kangaroos spent 74.6 ± 1.1% of the focal observations foraging. There was no interaction between treatment and observation period (*F*_1, 206.0_ = 0.38, *P* = 0.54). However, the proportion of time spent foraging was significantly affected by observation period (*F*_1, 213.84_ = 6.09, *P* = 0.01), with all females foraging less in the second period of focal observations. There were no effects of treatment group (*F*_1, 21.15_ = 0.77, *P* = 0.39, Fig 2), temperature (*F*_1, 217.12_ = 0.01, *P* = 0.91), time of day (*F*_1, 217.39_ = 1.65, *P* = 0.20), pasture height (*F*_2, 224.25_ = 1.38, *P* = 0.26), group size (*F*_1, 227.44_ = 0.27, *P* = 0.61) or nearest-neighbour distance (*F*_1, 222.14_ = 0.02, *P* = 0.89) on feeding duration.

### Chewing duration

Chewing occurred for 12.2 ± 0.54% of the time. There was no interaction between treatment and observation period (*F*_1, 206.07_ = 1.42, *P* = 0.24). There were also no effects of observation period (*F*_1, 212.03_ = 0.81, *P* = 0.37), treatment group (*F*_1, 22.0_ = 0.02, *P* = 0.89, Fig 2), temperature (*F*_1, 214.22_ = 0.98, *P* = 0.32), time of day (*F*_1, 214.33_ = 1.49, *P* = 0.22), pasture height (*F*_2, 225.24_ = 1.77, *P* = 0.17), group size (*F*_1, 224.35_ = 0.93, *P* = 0.33) or nearest-neighbour distance (*F*_1, 218.63_ = 0.20, *P* = 0.66) on chewing duration.

### Bite rates

Female kangaroos had a mean bite rate of 54.7 ± 0.72 bites per min. There was no interaction between treatment and observation period (*F*_1, 205.54_ = 0.21, *P* = 0.64). However, bite rates increased significantly with temperature (*F*_1, 210.32_ = 5.12, *P* = 0.03). There were no significant effects of treatment group (*F*_1, 21.30_ = 0.18, *P* = 0.67, Fig 2), observation period (*F*_1, 216.85_ = 1.67, *P* = 0.20), time of day (*F*_1, 211.23_ = 0.55, *P* = 0.46), pasture height (*F*_2, 220.83_ = 2.53, *P* = 0.08), group size (*F*_1, 217.08_ = 0.01, *P* = 0.90) or distance to nearest neighbour (*F*_1, 213.06_ = 0.79, *P* = 0.38) on bite rates.

### Bite-step ratio

On average, kangaroos took 49.9 ± 2.1 bites per step. There was no interaction between treatment and observation period (*F*_1, 208.96_ = 1.26, *P* = 0.26). However, bite-step ratio was affected by the distance to the focal kangaroo’s nearest neighbour (*F*_1, 219.33_ = 3.98, *P* = 0.05), increasing when neighbours were close. There was no effect of observation period (*F*_1, 219.72_ = 0.01, *P* = 0.93), treatment group (*F*_1, 21.34_ = 0.02, *P* = 0.90, Fig 2), temperature (*F*_1, 215.49_ = 0.04, *P* = 0.84), time of day (*F*_1, 216.66_ = 0.11, *P* = 0.72), pasture height (*F*_2, 223.09_ = 0.85, *P* = 0.43), or group size (*F*_1, 223.72_ = 0.38, *P* = 0.54) on bite-step ratios.

## Discussion

This study is among the first to experimentally test the influence of parasites on forage intake by a wild herbivore in a free-ranging context. We predicted that foraging would be adjusted as a mechanism to compensate for the costs of parasitism. However, contrary to our predictions, the experimental manipulation of parasite burdens did not result in changes in any of the key foraging variables we measured. Although factors such as an individual’s age and history of parasitism can influence parasite resistance and motivation to feed, the BACI design we used controlled for these individual differences. Our results suggest that, despite carrying large burdens of gastrointestinal parasites (relative to other herbivores [70]), the foraging strategy of female kangaroos is likely be driven by factors unrelated to parasitism. Alternatively, the kangaroos in our study system may have been foraging in a high nutrient environment, allowing them to receive sufficient nutrients to offset the costs of parasitism.

Previous studies have demonstrated that kangaroos have some degree of plasticity in their foraging behaviour, resulting in changes in forage intake [46, 48, 64]. The pasture at Anglesea is closely cropped and thus bite rate should have been a good surrogate measure for intake rate. The calculated bite rates (mean 54.7 ± 0.72 bites/min) from our observations were within the range reported (30–91.7 bites/min) for lactating female kangaroos at other sites [46, 48, 64]. However, there was no effect of anthelmintic treatment on bite rates in our study. Feeding duration was slightly reduced in the second observation period, suggesting that foraging was influenced more by extrinsic factors, such as resource availability or predation risk. Peak pasture growth occurs in spring (during the second observation period), which likely contributed to this finding. For example, Bennett’s wallabies (*Macropus rufogriseus bennettianus*) tend to reduce their foraging duration as pasture biomass increases [60]. This finding could also be a consequence of increased vigilance by mothers during peak lactation in spring, when their young are most vulnerable to predation by red foxes [71]. Our results suggest that female kangaroos may adopt a foraging strategy driven more by food availability or predation, than by parasites. Observations carried out at a different time of the year might yield different results.

Another explanation for our results is that infected female kangaroos might have employed alternative, unmeasured foraging adjustments to combat the costs of parasitism. An alternate foraging mechanism used by ruminants at a micro-foraging scale is a change in bite depth [28], which can alter food intake. However, if kangaroos were employing this tactic it is likely that chewing duration would have been different between the two groups in order to process extra material [72]. Alternatively, kangaroos with low parasite burdens may have altered the overall amount of time that they spent foraging, either throughout the day or at night. Kangaroos adjust their daytime foraging at the expense of time resting when the costs of reproduction are manipulated [46, 48], and Grant’s gazelles, *Nanger granti*, show adjustments in time-budgets in response to parasitism [73]. Unfortunately, we did not have the capacity to measure total daily foraging time in this experiment. It is also possible that kangaroos altered a suite of foraging variables simultaneously, with the low power for each of our variables making it difficult to detect change in one variable alone.

The results of our study are consistent with the findings of Jones *et al*. [74] and Fleurance *et al*. [75], who found no changes in foraging behaviour with parasitism. However, the majority of studies in domestic ruminants have shown that infection with gastrointestinal parasites actually has suppressive effects on food intake, for example with reductions of 30–60% in sheep [33, 58, 76]. Although the mechanisms driving these reductions are still being explored, this behaviour is currently considered to be an adaptive response by the host to avoid further intake of larvae [77]. Domestication has resulted in the modification of many behavioural traits via selection, including foraging motivation [78]. In contrast to free-ranging herbivores, domesticated ruminants are typically kept at high densities and subjected to high rates of parasite transmission. Free-ranging herbivores are arguably under stronger natural selective pressures, and their behaviours may be more likely to reflect the true fitness costs of foraging decisions [13, 27]. Indeed, studies on the same species under agricultural and free-ranging conditions often show disparities [78]. Therefore we did not expect reduced foraging motivation in infected kangaroos, but rather augmented forage intake to satisfy their increased energetic requirements [36], analogous to the costs of reproduction [46]. Despite this, there is some limited evidence for similar reductions in food intake in wild mammalian hosts, in herbivores such as red deer *Cervus elaphus* [79] and reindeer *Rangifer tarandus* [80], and in small mammals such as rats *Rattus norvegicus* [81] and Cape ground squirrels *Xerus inauris* [82].

Nutrition can play an important role in the resilience of hosts and their ability to withstand nematode infection [83, 84]. The pasture at Anglesea is irrigated, fertilized and regularly mown, which encourages new foliage with high protein content [85, 86]. Furthermore, high levels of faecal contamination tend to have a fertilizing effect on pasture, increasing the nutrient content of the sward [87]. At Anglesea, female kangaroos may be able to extract sufficient nutrients from their environment to offset any costs of parasitism. Unlike domestic sheep [88] for example, kangaroos may not need to make riskier grazing decisions. The habitat at Anglesea is still relatively complex compared to the studies carried out in controlled, agricultural systems [28, 34]. During the experiment, kangaroos remained free-ranging over a number of habitats, including remnant patches of native vegetation and a nearby, floristically-rich heathland, so there was potential for sward selection to occur outside the focal observations. Differences in foraging behaviour might only become apparent in low nutrient systems or in heavily parasitised hosts experiencing extreme conditions, such as drought.

The parasite burden of an individual will affect its foraging strategy only if it is able to respond to physiological stimuli in some way. The exact impacts of each of the helminth species infecting eastern grey kangaroos are unclear, but some pathological impacts have been described [89]. There are significant pathological changes associated with the larvae of one species, *Rugopharynx rosemariae*, which causes lesions on the gastric mucosa of kangaroos [90]. This tissue damage may cause significant disruptions to digestion and nutrient absorption in the host, and could take some time to resolve. This nematode species is present within the kangaroo population at our study site [41], but it is possible that kangaroos may not be able to evaluate any differences in their digestive efficiency in the short term. In reindeer, for example, reductions in food intake could only be detected six months following treatment [80]. In kangaroos, the interpretation of anthelmintic action is limited by inadequate information on the life cycles of the majority of the strongyle nematodes infecting these hosts. In particular, there is no information on the activity of the anthelmintic against hypobiotic larvae in tissues. We were also restricted to the use of a once-off anthelmintic dose in our experiment, due to the low efficacy of long-acting macrocyclic lactones in kangaroos [57]. In order to repeat this experiment over a longer time-scale, kangaroos would need to be recaptured and treated at least every three months, which would be logistically challenging. Future studies await the availability of long-acting anthelmintics suitable for kangaroos.

## Supporting Information

S1 Dataset All original data. Individual focal behavioural data.
(XLSX)

S2 Dataset All original data. Individual faecal egg count data.
(XLSX)
